# Comparative Analysis of Fungal Diversity in Rhizospheric Soil from Wild and Reintroduced *Magnolia sinica* Estimated via High-Throughput Sequencing

**DOI:** 10.3390/plants9050600

**Published:** 2020-05-08

**Authors:** Qingqing Shen, Junyu Yang, Daifa Su, Zhiying Li, Wei Xiao, Yongxia Wang, Xiaolong Cui

**Affiliations:** 1 Yunnan Institute of Microbiology, School of Life Sciences, Yunnan University, Kunming 650091, China; ynwsxysqq@163.com (Q.S.); junyuyang90@mail.ynu.edu.cn (J.Y.); daifasu@mail.ynu.edu.cn (D.S.); zyli@ynu.edu.cn (Z.L.); weixiao@ynu.edu.cn (W.X.); wangyx@ynu.edu.cn (Y.W.); 2 College of Environment and Resources, Wenshan University, Wenshan 663099, China; 3 State Key Laboratory for Conservation and Utilization of Bio-Resources in Yunnan, Yunnan University, Kunming 650091, China

**Keywords:** wild and reintroduced *Magnolia sinica*, rhizosphere, fungal community diversity, FUNGuild and function, soil attributes

## Abstract

*Magnolia sinica* is a critically endangered species and considered a “plant species with extremely small populations” (PSESP). It is an endemic species in southeastern Yunnan Province, China, with reproductive barriers. Rhizosphere fungi play a crucial role in plant growth and health. However, the composition, diversity, and function of fungal communities in wild and reintroduced *M. sinica* rhizospheres remain unknown. In this study, Illumina sequencing of the internal transcribed spacer 2 (ITS2) region was used to analyze rhizospheric soil samples from wild and reintroduced *M. sinica*. Thirteen phyla, 45 classes, 105 orders, 232 families, and 433 genera of fungi were detected. Basidiomycota and Ascomycota were dominant across all samples. The fungal community composition was similar between the wild and reintroduced rhizospheres, but the fungal taxa relative abundances differed. The fungal community richness was higher in the reintroduced rhizosphere than in the wild rhizosphere, but the diversity showed the opposite pattern. Soil nutrients and leaf litter significantly affected the fungal community composition and functional diversity. Here, the composition, structure, diversity, and ecological functions of the fungal communities in the rhizospheres of wild and reintroduced *M. sinica* were elucidated for the first time, laying a foundation for future research and endangered species protection.

## 1. Introduction

The rhizosphere is a narrow area of soil surrounding the root of a plant and is highly influenced by plant root exudates. A large number of plant-associated microbial communities [1,2], including bacteria, fungi, archaea, protozoa, and other microorganisms with unknown functions, live in this soil area. Therefore, these microbial communities are considered the second genome of the plant and are essential to plant health [3]. The rhizosphere is a crucial factor affecting relationships among plants, soils and microorganisms; it is also the most active region for material and energy exchange and plays an essential role in nutrient circulation in the ecosystem [1,4]. Fungi originated one billion years ago and are widely distributed in terrestrial ecosystems and water environments, with 3.8 million known species [5]. Fungi have a variety of methods to obtain nutrients, from saprophytic to symbiotic to parasitic methods, and can even switch between them [6]. Rhizosphere fungi are essential components of soil-plant ecosystems. The composition and structure of fungal communities are closely related to plant growth and development and the transmission and development of soil-borne diseases [7]. Fungi can also have symbiotic or pathogenic relationships with plants and animals and play an essential role in soil nitrogen transformation, carbon fixation, organic matter (OM) decomposition, and nutrient cycling [8]. The diversity and structure of the soil fungal community can also be used as a biological indicator of soil health [9]. Therefore, the community diversity and function of fungi in rhizospheric soil has become a hot topic in microbial molecular ecology research. Previous studies have shown that the dominant fungi at the phylum level in the rhizosphere are Ascomycota and Basidiomycota and that the most common genera are *Fusarium*, *Penicillium*, and *Aspergillus* [10,11].

As a member of the family Magnoliaceae, *Magnolia sinica* (Law) Noot. is closely related to species of the genus *Manglietia* [12]. This species is considered to be the most primitive of the Magnoliaceae and is an ideal landscaping and high-quality timber species. In 1979, it was first named as *Manglietiastrum sinicum* (Law) by the Chinese botanist Law. Subsequently, botanists split the species into *Magnolia sinicum*, Huagaimu [13,14], *Manglietia sinica* [15], and *Pachylarnax sinica* [16]. This species is referred to as *Magnolia sinica* in this study because of its status as an internationally widely used and accepted name (from the website www.theplantlist.org). According to previous reports, *M. sinica* is an endemic species in southeastern Yunnan Province, and only 52 wild individuals and eight populations have been found in the natural environment [12,16]. Propagation by seed is essential for the reproduction of this species. However, the deterioration of suitable habitats, overexploitation of the soil, limited dispersal of seeds (with birds as the mode of dispersal), low seed germinability, and other factors have ultimately led to the narrow distribution and extremely small number of populations of *M. sinica* [17,18]. As a result, the Chinese State Report on Biodiversity Editorial Committee listed *M. sinica* as a critically endangered species in the China Plant Red Book and identified it as a “plant species with extremely small populations (PSESP)” [19,20]. To date, researchers have mainly focused on seed germination [18], reintroduction and cultivation [14], and the separation of compounds from plants [21]. For example, seeds treated with moist chilling have the highest germination rate, and compounds isolated from plants show the inhibition of platelet aggregation, antimicrobial activity, and weak cytotoxic activity against human tumor cells [22]. Reintroduced indicates that tree seedlings were replanted in their native habitat or a similar habitat, rather than in a habitat where the plant had previously grown but subsequently disappeared. Its seeds and seedlings are rarely found in the wild. Furthermore, reintroduction is essential for the adequate protection of *M. sinica* plants. Our research will provide insight into wild and reintroduced plants from the perspective of the plant microbiome; these results will help to protect endangered plants, promote plant growth, prevent the occurrence of diseases and insect pests, and provide a theoretical basis for the protection of endangered plants.

*M. sinica* has been identified as an endangered plant of “first-grade state protection” in China [19], and research on its rhizospheric soil fungal diversity has not yet been reported. Therefore, this study used an Illumina MiSeq platform to compare the composition, diversity, and structure of fungi in rhizospheric soil between wild and reintroduced *M. sinica* for the first time. The FUNGuild database was also used to analyze and identify the nutrient types and functions of the fungi [23]. The relationships between the fungal communities and soil physicochemical factors were analyzed to provide a theoretical basis for the isolation and screening of symbiotic fungi and to guide research on the conservation of *M. sinica*.

## 2. Results

### 2.1. Variation in Rhizospheric Soil Properties

As shown in Table 1, independent-sample *t*-tests were performed to analyze the physical and chemical property data of the rhizospheric soil from the wild and reintroduced *M. sinica*. The results showed that the rhizospheric soil from the two groups was weakly acidic. Furthermore, the pH value of the rhizospheric soil from the reintroduced plants was significantly higher than that from the wild *M. sinica* (T = 31.54, *P* < 0.01). The OM content (T = 68.71, *P* < 0.01), HN (T = 295, *P* < 0.01), TN = 128.35, *P* < 0.01), TK (T = 55.01, *P* < 0.01), and EC (T = 47.45, *P* < 0.01) in the rhizospheric soil from the wild plants were significantly higher than those in the rhizospheric soil from the reintroduced plants. However, the value of AK (T = −35.36, *P* < 0.01) in the rhizospheric soil from the wild plants was significantly lower than that in the rhizospheric soil from the reintroduced plants. The TP (*P* > 0.05) and TWSS (T = 2.12, *P* = 0.101 > 0.05) did not significantly differ between the two groups. Overall, the nutrient content of the rhizospheric soil from the wild plants was higher than that of the rhizospheric soil from the reintroduced plants.

### 2.2. Evaluation of Sequencing Quality

**Figure 1:**
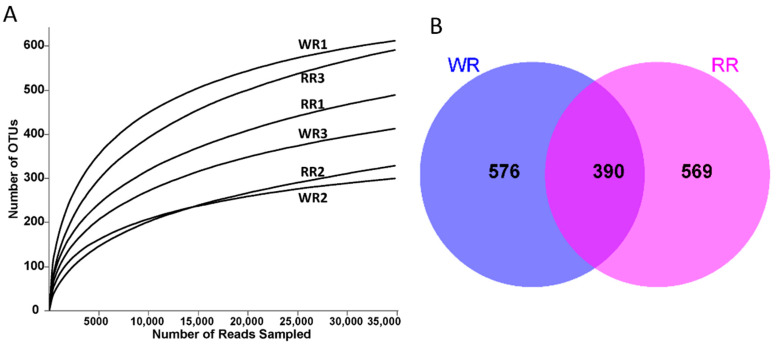
(**A**). Rarefaction curves of rhizospheric soil samples from wild and reintroduced *M. sinica* at a 97% similarity threshold; (**B**). OUT-Venn graph of rhizospheric soil samples from wild and reintroduced *M. sinica* (WR1, WR2, and WR3 represent the three replicates of rhizospheric soil samples from wild plants (WR); RR1, RR2, and RR3 represent the three replicates of rhizospheric soil samples from reintroduced plants (RR)).

A total of 359,936 ITS sequences were obtained by high-throughput sequencing of the six rhizospheric soil samples from the wild and reintroduced *M. sinica*. After filtering and optimizing the ITS sequences, 344,898 valid sequences were ultimately obtained (Table 2). At the 97% similarity level, subsampling the number of sequences from all samples based on the minimum threshold resulted in 34,720 sequences from the RR2 sample. Then, a total of 2728 OTUs were obtained for annotation and classification through annotation and clustering; the total numbers of OTUs of rhizospheric soil fungi associated with the wild and reintroduced *M. sinica* were 1322 (*n* = 3) and 1406 (*n* = 3), respectively. Figure 1A shows the rarefaction curves of the numbers of fungal OTUs in all the samples of rhizospheric soil. When the curves of the numbers of OTUs in all samples begin to flatten, the results indicate that the sequencing depth of all rhizospheric soil samples is reasonable and can truly and comprehensively reflect the structure and composition of the fungal community in the rhizospheric soil samples from the wild and reintroduced *M. sinica*. In addition, the coverage index values for all samples presented in Table 2 are higher than 99.5%, which also indicates that the sequencing depth of all samples in the present study is reasonable and that further results are reliable [4].

### 2.3. Alpha-Diversity Analysis of Rhizospheric Soil Samples from Wild and Reintroduced M. sinica

Alpha diversity (α-diversity) reflects the richness and diversity of the fungal communities in rhizospheric soil samples from wild and reintroduced *M. sinica*. As shown in Table 2, the richness index values (Sobs, ACE, and Chao) were greater in the reintroduced rhizospheric soil than in the wild rhizospheric soil, indicating that the richness of fungal communities in the reintroduced rhizospheric soil was higher than that in the wild rhizospheric soil. In addition, Shannon and Simpson index values demonstrated that the diversity of fungal communities in the wild rhizospheric soil was higher than that in the reintroduced rhizospheric soil. However, all the index values mentioned above did not significantly differ (*P* > 0.05). The OUT-Venn graph directly reflects the common and unique OTUs in the wild and reintroduced samples. Therefore, according to Figure 1B, a total of 1535 different types of OTUs were obtained from the wild and reintroduced samples, among which 390 common types of OTUs were observed. The number of unique types of OTUs from the wild samples was 576 and that from the reintroduced samples was 569; thus, the total number of types of OTUs in the wild samples was higher than that in the reintroduced samples. In summary, based on the alpha-diversity index analysis and OUT-Venn graph, the fungal communities in both the wild and reintroduced rhizospheric soil samples were extremely species-rich and similar, but there were some differences in their structure and the relative abundances of fungal taxa.

### 2.4. Composition and Structure of the Fungal Communities in the Rhizospheric Soil of Wild and Reintroduced M. sinica

**Figure 2:**
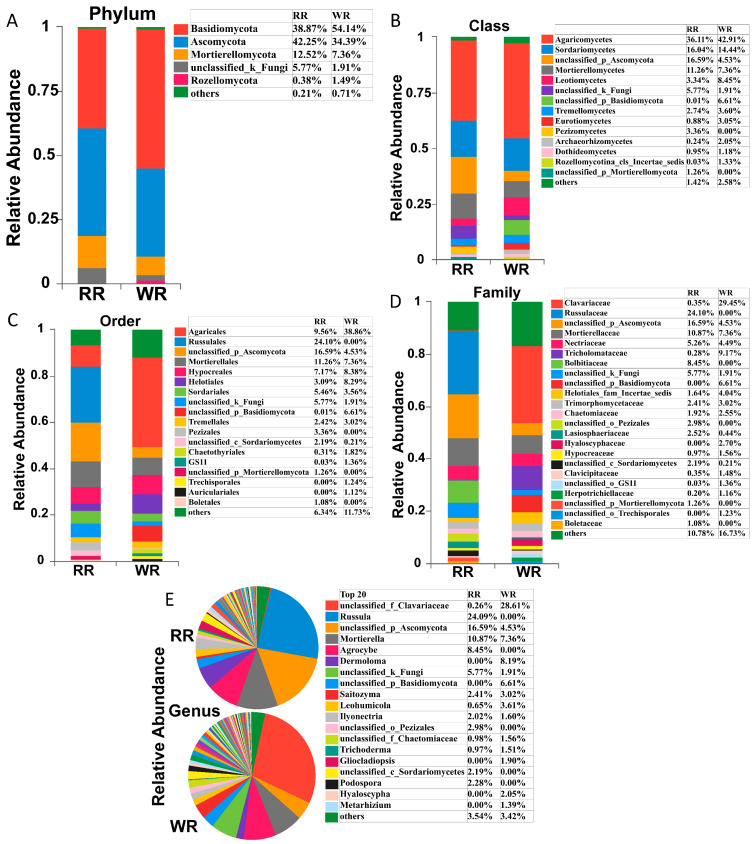
Relative abundances of fungal taxa in rhizospheric soil samples from wild and reintroduced *M. sinica* at the phylum (**A**, all phyla with relative abundance greater than 1%), class (**B**, all classes with relative abundance greater than 1%), order (**C**, all orders with relative abundance greater than 1%), family (**D**, all families with relative abundance greater than 1%), and genus (**E**, the top 20 most abundant genera) levels. The remaining members with a relative abundance of <1% are denoted as “others” (rhizospheric soil samples from wild plants (WR, *n* = 3); rhizospheric soil samples from reintroduced plants (RR, *n* = 3)).

High-throughput sequencing identified a total of 13 phyla, 45 classes, 105 orders, 232 families, and 433 genera in the six samples of rhizospheric soil from wild and reintroduced *M. sinica*. Figure 2 shows that the compositions of the rhizospheric soil fungal communities were similar between the wild and reintroduced plants, but the relative abundances of the fungal taxa were significantly different. The main fungal phyla in the rhizospheric soil of both the wild and reintroduced plants were Basidiomycota, Ascomycota, Mortierellomycota, unclassified groups, and Rozellomycota (Figure 2A). Among them, Basidiomycota was the dominant phylum in the wild rhizospheric soil, with a relative abundance of 54.14%, which was higher than the relative abundance of this phylum, at 38.87%, in the reintroduced rhizospheric soil. Ascomycota was the dominant phylum in the reintroduced rhizospheric soil, with a relative abundance of 42.25%, which was higher than that in the wild rhizospheric soil (34.39%). Other phyla had lower relative abundance values.

Agaricomycetes was the most abundant fungal class, with a relative abundance of 42.91%, followed by Sordariomycetes, with a relative abundance of 14.44%, and Leotiomycetes, with a relative abundance of 8.45% (Figure 2B). However, in the reintroduced rhizospheric soil, Agaricomycetes was the most abundant fungal class, with a relative abundance of 36.11%, followed by an unclassified (Ascomycota) group, with 16.59%, and Sordariomycetes, with 16.04%. It is worth noting that Pezizomycetes was found in only the reintroduced rhizospheric soil, with a relative abundance of 3.36%, while the relative abundance of Leotiomycetes in the wild rhizospheric soil was 2.53 times that in the reintroduced soil, and the relative abundance of Eurotiomycetes in the wild rhizospheric soil (relative abundance = 3.05%) was 3.47 times that in the reintroduced rhizospheric soil (0.88%). Therefore, there were significant differences in the relative abundance of the fungal taxa in the wild and reintroduced rhizospheric soil at the class level.

The order with the highest relative abundance in the wild rhizospheric soil was Agaricales, reaching 38.86%, which was 4.06 times higher than that in the reintroduced rhizospheric soil (9.56%) (Figure 2C). The second most abundant fungal group was Hypocreales, with a relative abundance of 8.38%, which was higher than that in the reintroduced rhizospheric soil. The third most abundant fungal group was Helotiales (8.29%), with a value of 2.68 times higher than that in the reintroduced rhizospheric soil. The highest relative abundance in the reintroduced rhizospheric soil was observed for Russulales (24.1%), followed by Mortierellales (11.26%) and Agaricales (9.56%). It is worth noting that Chaetothyriales had low relative abundance in both the wild and reintroduced rhizospheric soil, but its relative abundance in the wild rhizospheric soil (1.82%) was 5.97 times greater than that in the reintroduced rhizospheric soil (0.31%). Interestingly, Russulales, Pezizales (3.36%), and Boletales (1.08%) occurred in only the reintroduced rhizospheric soil, while Trechisporales (1.24%) and Auriculariales (1.12%) were found in only the wild rhizospheric soil.

At the family level (Figure 2D), Clavariaceae was the dominant group in the wild rhizospheric soil, with a relative abundance of 29.45%, which was greater than that in the reintroduced rhizospheric soil (0.35%), followed by Tricholomataceae and Mortierellaceae, with relative abundances of 9.17% and 7.36%, respectively. Moreover, the relative abundance of Tricholomataceae in the wild rhizospheric soil was 32.75 times higher than that in the reintroduced rhizospheric soil. However, in the reintroduced rhizospheric soil, the dominant group with the highest relative abundance was Russulaceae (24.1%), followed by Mortierellaceae (10.87%), and Bolbitiaceae (8.45%). Notably, the relative abundance of Lasiosphaeriaceae in the reintroduced rhizospheric soil was 5.73 times higher than that in the wild rhizospheric soil, and the relative abundances of Hypocreaceae and Herpotrichiellaceae in the wild rhizospheric soil were 1.61 and 5.8 times higher than those in the reintroduced rhizospheric soil, respectively. Interestingly, Russulaceae, Bolbitiaceae, an unclassified (Pezizales) group, and another unclassified (Mortierellomycota) group were found in only the reintroduced rhizospheric soil, while an unclassified (Basidiomycota) group, Hyaloscyphaceae, and another unclassified (Trechisporales) group occurred in only the wild rhizospheric soil.

As shown in Figure 2E, at the genus level, the dominant fungal groups in terms of relative abundance in the wild and reintroduced rhizosphere soils included an unclassified group, *Russula*, *Mortierella*, *Agrocybe*, *Dermoloma*, *Saitozyma*, *Leohumicola*, *Ilyonectria*, *Trichoderma*, *Gliocladiopsis*, *Podospora*, *Hyaloscypha,* and *Metarhizium*. Among them, *Russula*, *Agrocybe*, and *Podospora* were found in only the reintroduced rhizospheric soil, with relative abundances of 24.09%, 8.45%, and 2.28%, respectively. However, *Dermoloma*, *Gliocladiopsis*, *Hyaloscypha*, and *Metarhizium* were found in only the wild rhizospheric soil, with relative abundances of 8.19%, 1.9%, 2.05%, and 1.39%, respectively. It is worth noting that the relative abundance of *Leohumicola* in the wild rhizospheric soil (3.61%) was 5.55 times higher than that in the reintroduced rhizospheric soil (0.65%). The dominant groups in terms of relative abundance in the wild rhizospheric soil were an unclassified (Clavariaceae) group, *Mortierella,* and *Dermoloma*, while the dominant groups in the reintroduced rhizospheric soils were *Russula*, *Mortierella*, and *Agrocybe*, among which *Mortierella* had the highest relative abundance in both the wild and the reintroduced rhizospheric soil.

### 2.5. Comparative Analysis of the Fungal Communities in the Rhizospheric Soil of Wild and Reintroduced M. sinica

**Figure 3:**
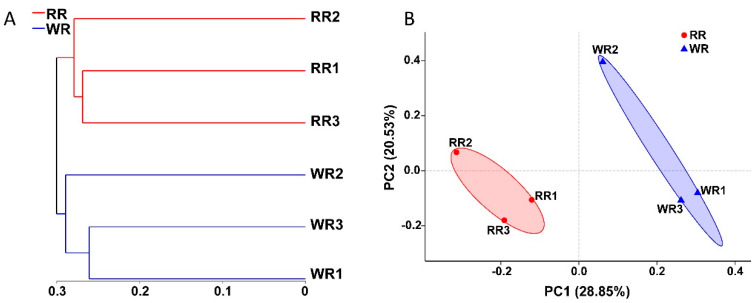
Hierarchical clustering tree (**A**) and principal coordinates analysis (PCoA) results (**B**) for the fungal communities in rhizospheric soil from wild and reintroduced *M. sinica* at the OTU level using the unweighted UniFrac method (WR1, WR2, and WR3 represent the three replicates of rhizospheric soil samples from wild plants (WR); RR1, RR2, and RR3 represent the three replicates of rhizospheric soil samples from reintroduced plants (RR)).

In this study, the fungal communities in the three samples of wild rhizospheric soil and three samples of reintroduced rhizospheric soil were compared. As shown in Figure 3A, according to the hierarchical clustering analysis, a clustering tree was constructed with the OTUs of the six samples using the unweighted UniFrac algorithm. The three wild samples and three reintroduced samples clustered together, indicating the presence of significant differences between the wild and reintroduced rhizospheric soil fungal communities. In addition, principal coordinate analysis (PCoA) was used to compare the wild and reintroduced rhizospheric soil fungal communities (Figure 3B). The results based on unweighted UniFrac distances showed that the contributions of PC1 and PC2 were 28.85% and 20.53%, respectively. The wild and reintroduced rhizospheric soil samples also clustered together, indicating that there were significant differences in the rhizospheric soil fungal communities between the wild and reintroduced *M. sinica*.

**Figure 4:**
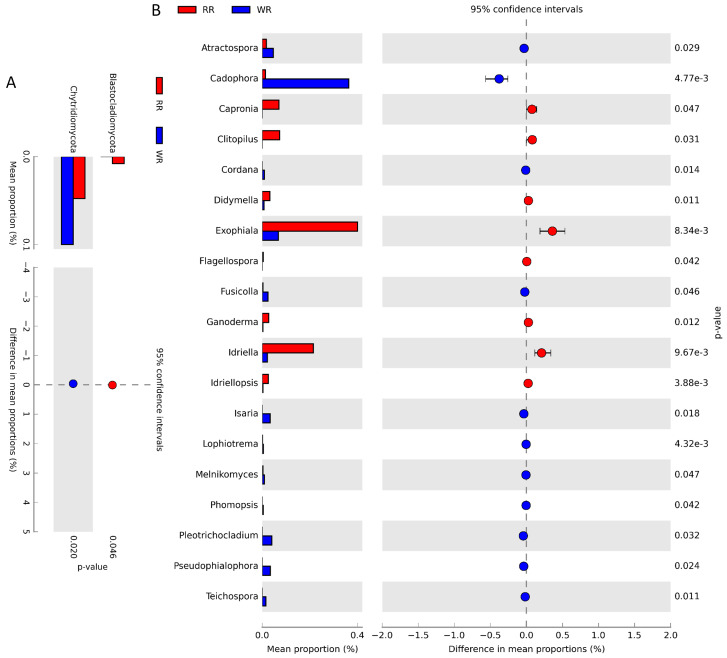
Comparison of fungal abundance in rhizospheric soil from wild (WR) and reintroduced (RR) *M. sinica* using White’s non-parametric *t*-test at the phylum (**A**) and genus (**B**) level (rhizospheric soil samples from wild plants (WR, *n* = 3); rhizospheric soil samples from reintroduced plants (RR, *n* = 3).

The differences in fungal community structures between the wild and reintroduced rhizospheric soils were also analyzed with White’s non-parametric *t*-test. As shown in Figure 4A, there were significant differences in the relative abundances of taxa at the phylum level. The relative abundance of Blastocladiomycota (*P* = 0.046, <0.05) in the reintroduced rhizospheric soil was significantly greater than that in the wild rhizospheric soil, and the relative abundance of Chytridiomycota (*P* = 0.02, <0.05) in the wild rhizospheric soil was significantly higher than that in the reintroduced rhizospheric soil. At the genus level (Figure 4B), the relative abundances of *Atractospora* (*P* = 0.029, <0.05), *Cadophora* (*P* < 0.01), *Capronia* (*P* = 0.047, <0.05), *Clitopilus* (*P* = 0.031, <0.05), *Cordana* (*P* = 0.014, <0.05), *Didymella* (*P* = 0.011, <0.05), *Exophiala* (*P* <0.01), *Flagellospora* (*P* = 0.042, <0.05), *Fusicolla* (*P* = 0.046, <0.05), *Ganoderma* (*P* =0.012, <0.05), *Idriella* (*P* < 0.01), *Idriellopsis* (*P* < 0.01), *Isaria* (*P* = 0.018, <0.05), *Lophiostoma* (*P* < 0.01), *Melnikomyces* (*P* = 0.047, <0.05), *Phomopsis* (*P* = 0.042, <0.05), *Plectosphaerella* (*P* = 0.032, <0.05), *Pseudocercospora* (*P* = 0.024, <0.05), and *Teichospora* (*P* = 0.011, <0.05) were significantly different between the wild and reintroduced rhizospheric soil. Among them, the relative abundances of *Atractospora*, *Cadophora*, *Cordana*, *Fusicolla, Isaria*, *Lophiostoma*, *Melnikomyces*, *Phomopsis*, *Plectosphaerella*, *Pseudocercospora*, and *Teichospora* were significantly greater in the wild rhizospheric soil than in the reintroduced rhizospheric soil, while the relative abundances of the other genera were less than those in the reintroduced rhizospheric soil.

### 2.6. Correlations between Fungal Diversity and Rhizospheric Soil Physicochemical Properties

**Figure 5:**
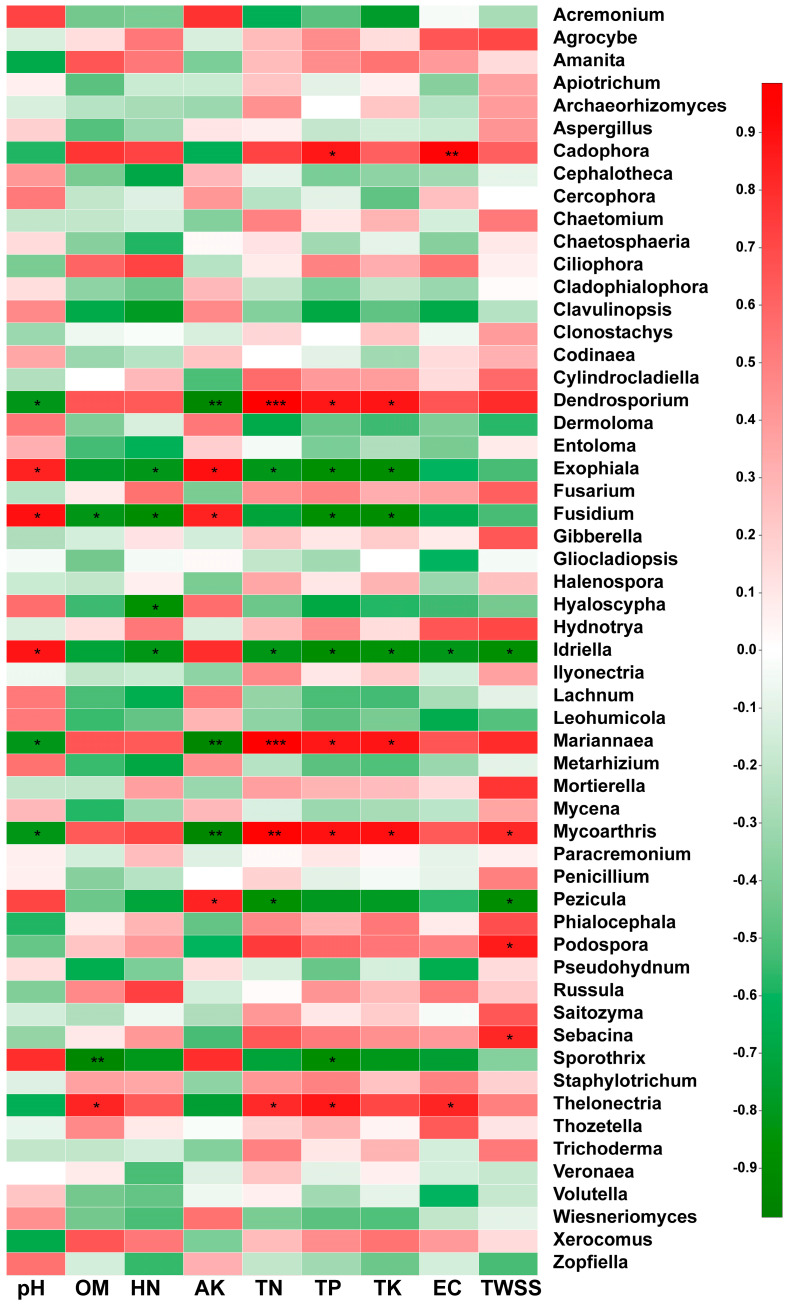
Spearman analysis of the relationships between soil physicochemical properties and the most abundant fungal genera in the rhizospheric soil of wild (WR) and reintroduced (RR) *M. sinica* at the genus level (rhizospheric soil samples from wild plants (WR, *n* = 3); rhizospheric soil samples from reintroduced plants (RR, *n* = 3); * 0.01 < *P* ≤ 0.05, ** 0.001 < *P* ≤ 0.01, *** *P* ≤ 0.001.

In this study, Spearman analysis was used to evaluate the correlations between soil physicochemical properties and the relative abundances of rhizosphere soil fungi at the genus level. According to the results shown in Figure 5, the pH value was significantly negatively correlated with the relative abundances of *Dendrostorium*, *Mariannaea*, and *Mycoarthris* and significantly positively correlated with the relative abundances of *Exophiala*, *Fusidium*, and *Idriella*. The OM content showed a significant positive correlation with *Thelonectria*, a significant negative correlation with *Sporothrix* and *Fusidium*, and no significant correlation with the other fungal genera. The HN was significantly negatively correlated with only *Exophiala*, *Fusidium*, *Hyaloscypha*, and *Idriella*. The AK was significantly positively correlated with *Pezicula*, *Exophiala*, and *Fusidium* but significantly negatively correlated with *Dendrasporium*, *Mariannaea*, and *Mycoarthris*. The TN was significantly positively correlated with *Thelonectria*, *Mariannaea*, *Mycoarthris*, and *Dendroporium* but significantly negatively correlated with *Exophiala*, *Idriella*, and *Pezicula*. The TP was significantly positively correlated with *Thelonectria*, *Mycoarthris*, *Mariannaea*, *Dendroporium,* and *Cadophora* but significantly negatively correlated with *Exophiala*, *Fusidium*, *Idriella,* and *Sporothrix*. The TK was significantly positively correlated with *Mycoarthris*, *Mariannaea*, and *Dendrostorium* but significantly negatively correlated with *Exophiala*, *Fusidium*, and *Idriella*. The EC was significantly positively correlated with only *Cadophora* and *Thelonectria* but significantly negatively correlated with only *Idriella*. The TWSS was significantly positively correlated with the genera *Sebacina*, *Podospora*, and *Mycoarthris* and significantly negatively correlated with the genera *Idriella* and *Pezicula*.

Overall, the physicochemical properties of soil pH, AK, TN, TP, and TK were found to have the most significant influence on the fungal communities, as these properties had a significant correlation with more than six fungal genera (*P* < 0.01).

### 2.7. Functional Prediction of the Fungi in the Rhizospheric Soil of Wild and Reintroduced M. sinica

**Figure 6:**
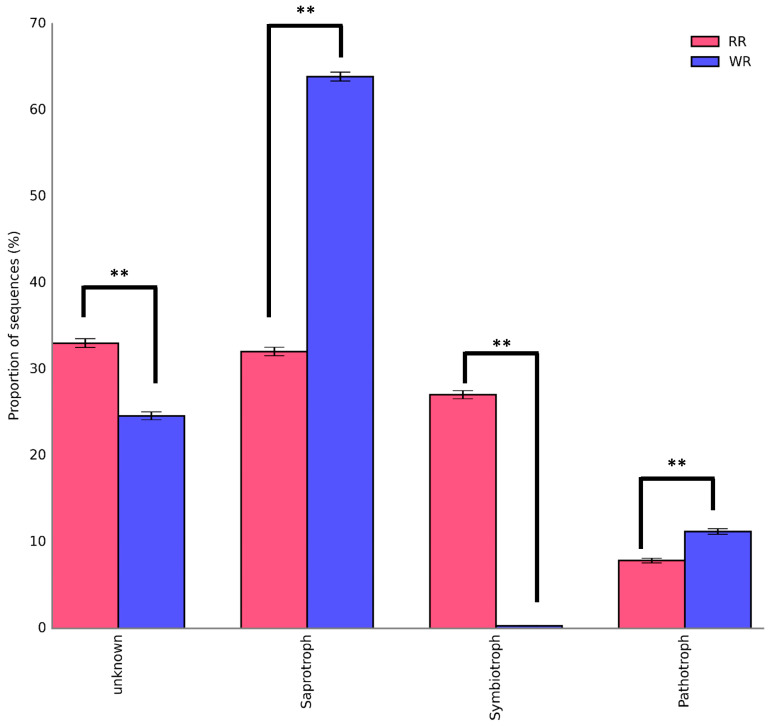
Trophic composition of rhizospheric fungal communities associated with wild and reintroduced *M. sinica* (rhizospheric soil samples from wild plants (WR, *n* = 3); rhizospheric soil samples from reintroduced plants (RR, *n* = 3)). ** *P* < 0.01.

The sequences of the fungi isolated from the wild and reintroduced rhizospheric soil were compared with those in the FUNGuild database to predict and analyze the differences in their functions [24,25]. Figure 6 shows the trophic modes of the fungi in the wild and reintroduced rhizospheric soils. The results demonstrate that most of the fungi in the wild rhizosphere soil were saprotroph (63.88%), followed by pathotroph, with a percentage of 11.2%, while symbiotroph accounted for only 0.31%. The composition and structure of the three trophic modes of fungi in the reintroduced rhizospheric soil differed as follows: saprotroph (32.06%) > symbiotroph (27.04%) > pathotroph (7.86%). The most important results were that the percentages of saprotroph and pathotroph in the wild rhizospheric soil were significantly higher than those in the reintroduced rhizospheric soil (*P* < 0.01). In addition, the percentage of symbiotroph in the reintroduced rhizospheric soil was significantly higher than that in the wild rhizospheric soil (*P* < 0.01). The results also showed large numbers of fungal taxa with an unidentified trophic mode in the wild and reintroduced rhizosphere soils (defined as having an unknown trophic mode). The proportion of fungal taxa with an unknown trophic mode (33.03%) was significantly higher in the reintroduced rhizospheric soils than in the wild rhizospheric soil (*P* < 0.01).

**Figure 7:**
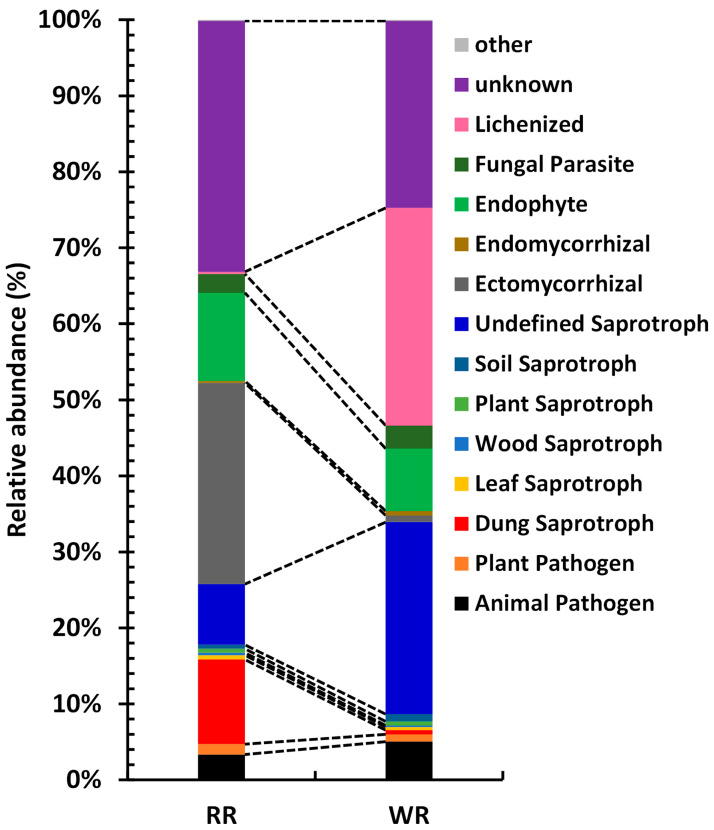
Functions of rhizospheric fungal associated with wild and reintroduced *M. sinica* (WR1, WR2, and WR3 represent the three replicates of rhizospheric soil samples from wild plants (WR); RR1, RR2, and RR3 represent the three replicates of rhizospheric soil samples from reintroduced plants (RR)).

The fungal taxa in the communities of the rhizospheres of wild and reintroduced *M. sinica* were divided into different functional groups according to their utilization of different environmental resources. A total of 15 functional groups were identified in this study (Figure 7), including Animal Pathogen, Plant Pathogen, Dung Saprotroph, Leaf Saprotroph, Wood Saprotroph, Plant Saprotroph, Soil Saprotroph, Undefined Saprotroph, Ectomycorrhizal, Endomycorrhizal, Endophyte, Fungal Parasite, Lichenized, Unknown and other groups (relative abundance <0.1%). With the exception of other groups and Plant Saprotroph, these functional groups showed significant differences in the rhizospheric soils of wild and reintroduced *M. sinica* (*P* < 0.01). The results show that the relative abundance of Lichenized fungi was the highest in the wild rhizospheric soil, reaching 28.61%, which was significantly greater than that in the reintroduced rhizospheric soil (relative abundance = 0.27%; *P* < 0.01), followed by Undefined Saprotroph, with a relative abundance of 25.26%, which was also significantly higher than that in the reintroduced rhizospheric soil (7.98%; *P* < 0.01). However, the relative abundance of Ectomycorrhizal fungi was highest in the reintroduced rhizospheric soil, accounting for 26.48% of the total, which was significantly higher than that in the wild rhizospheric soil (0.89%; *P* < 0.01), followed by Endophytes, with a relative abundance of 11.57%, which was also significantly higher than that in the wild rhizospheric soil (8.22%; *P* < 0.01). Both the wild and reintroduced rhizospheric soils hosted a large number of unknown groups, and the relative abundance in the reintroduced rhizospheric soil (33.04%) was significantly higher than that in the wild rhizospheric soil (24.61%; *P* < 0.01). In this study, Animal Pathogens and Plant Pathogens were predicted to occur in both the wild and reintroduced rhizospheric soils. The relative abundance of Animal Pathogens in the wild rhizospheric soil was 5.03%, which was significantly higher than that in the reintroduced rhizospheric soil (3.33%), and the relative abundance of Plant Pathogens in the reintroduced rhizospheric soil (1.38%) was significantly higher than that in the wild rhizospheric soil (0.95%; *P* < 0.01).

## 3. Discussion

In this study, high-throughput sequencing technology was used to explore the fungal diversity and community compositions in the rhizospheres soil of wild and reintroduced *M. sinica*. Our results showed that Basidiomycota, Ascomycota, Mortierellomycota, unclassified_k_Fungi, and Rozellomycota were the dominant fungal groups in the rhizosphere of wild and reintroduced plants, which is similar to previous results [26,27]. Previous studies have shown that fungi are important decomposers in ecosystems and can effectively decompose cellulose and lignin [7,10]. Among them, members of the phylum Ascomycota play an essential role in decomposing plant residues and degrading OM in the soil [28]. In this study, the OM content in the wild rhizosphere soil was 145.24 ± 1.27 g/kg, which was significantly greater than that in the reintroduced rhizosphere soil (64.03 ± 1.6 g/kg; *P* < 0.01). However, the relative abundance of Ascomycota was greater in the reintroduced rhizospheric soil (42.25%) than in the wild rhizospheric soil (34.39%), indicating that the large number of Ascomycota in the reintroduced rhizospheric soil promoted the decomposition of OM. When we collected the samples, we found that there was a large number of fallen leaves and a large amount of litter where the wild plants were growing, whereas there was only a small amount of litter associated with the reintroduced *M. sinica*. Interestingly, the results showed that the relative abundance of Basidiomycota in the wild plant rhizosphere (54.14%) was significantly greater than that in the reintroduced plant rhizosphere (38.87%). Furthermore, Basidiomycota can efficiently decompose lignocellulose, indicating that members of this taxon can decompose large amounts of leaves and litter from *M. sinica*. Therefore, our results were consistent with the findings of previous reports [27,28]. The genus *Mortierella* is common in soil; it has a strong ability to decompose cellulose and special ecological functions, such as promoting the absorption of minerals by plant roots and inhibiting pathogenic microorganisms [29,30]. The results of this study also showed that the relative abundance of *Mortierella* in the reintroduced plant rhizosphere (10.87%) was higher than that in the wild plant rhizosphere (7.36%), suggesting that this genus promoted the absorption of mineral elements by the roots of the reintroduced *M. sinica*, resulting in significantly lower TN, TK, and TP in the reintroduced rhizospheric soil than in the wild rhizospheric soil. Many studies have shown that the genus *Trichoderma* can control plant diseases, degrade OM, and promote plant growth [31,32]. Interestingly, we also found that the relative abundance of *Trichoderma* in the wild plant rhizosphere (1.51%) was 1.56 times higher than that in the reintroduced plant rhizosphere (0.97%), indicating that there was a large number of antagonistic fungi in the wild plant rhizosphere, potentially indicating the existence of species that can be screened for use in biocontrol programs.

We also found that the values of diversity indexes (such as the Sobs, ACE, and Chao indexes), which reflect the richness of fungal communities, were lower in the wild rhizospheric soil than in the reintroduced rhizospheric soil, indicating that the richness of the fungal communities was lower in the wild plant rhizosphere than in the reintroduced plant rhizosphere. However, the OM content was higher in the wild rhizospheric soil than in the reintroduced rhizospheric soil. Therefore, the results of our study are consistent with those showing that the richness of soil fungal communities decreased with an increase in soil OM content at a small local scale [33]. Previous studies have shown that soil fungi and plants can form symbiotic relationships, and a low OM content in the soil can promote the increased richness of fungal communities so that the plants can adapt to adverse environmental conditions [10,25]. Similarly, the diversity of plant community traits can also affect the complexity of soil microhabitats, which may cause the richness of soil fungal communities to change [34]. When we collected rhizospheric soil samples, we investigated the DBH of the wild and reintroduced plants and found that the DBH of the wild plants was significantly larger than that of the reintroduced plants and that the height of the wild plants was also significantly greater than that of the reintroduced plants. Therefore, the differences in height between the wild and reintroduced plants also led to differences in the richness of the soil fungal communities. Previous studies demonstrated that soil fungal diversity and community compositions were influenced by soil physical and chemical properties and plant diversity [24]. For example, the soil fungal diversity associated with wild rubber plants was higher than that in rubber plantations. Similarly, our results also indicated that the diversity of fungal communities in the wild plants was higher than that in the rhizosphere of reintroduced plants.

Changes in soil properties and nutrient content can affect the diversity and composition of microbial communities in the soil [24]. The analysis of the heatmap generated in this study showed that the physicochemical properties of soil pH, AK, TN, TP, and TK had the most significant influences on the fungal communities. Therefore, our results are consistent with the results of previous studies [35,36], indicating that soil pH and nutrient content are important factors influencing the fungal communities associated with wild and reintroduced *M. sinica*. According to the means by which they obtain nutrition, fungi can be divided into three trophic modes: pathotroph, saprotroph, and symbiotroph [23]. Saprotrophic fungi obtain nutrients by decomposing dead host cells, symbiotroph obtain nutrients by exchanging resources with host cells, and pathotroph acquire nutrients by harming host cells [37]. The results of this study show that most of the fungi in the wild rhizospheric soil were saprotroph, followed by pathotroph, and the proportion of symbiotroph was small. However, most fungi in the reintroduced rhizospheric soil were saprotroph, followed by symbiotroph. Previous studies have shown that the genus *Russula* accounts for the most species in Russulaceae; this genus has a significant economic value, is edible and medicinal, and has a symbiotic ectomycorrhizal relationship with trees [36,37]. Our results showed that the genus *Russula* was found in only the reintroduced rhizospheric soil, with a relative abundance of 24.09%. Therefore, the structure and function of the fungal community were consistent, indicating that our results were reliable. The fungal groups in the wild rhizospheric soil mainly obtained nutrients by decomposing litter, while those in the reintroduced rhizosphere soil mainly obtained nutrients by exchanging material with plant cells and decomposing dead host cells. Therefore, the litter near the wild and reintroduced *M. sinica* plants influenced the functional diversity of the rhizosphere fungal communities. A statistical analysis of the functional fungal groups was also conducted and showed that the relative abundance of plant pathogens in the wild plant rhizosphere was significantly less than that in the reintroduced plant rhizosphere (*P* < 0.01), indicating that there were a large number of antagonistic microorganisms in the wild rhizospheric soil and that biocontrol microorganisms could be obtained by culture-dependent methods. Our results also showed that there were a large number of unknown functional groups in the wild and reintroduced rhizosphere soil. Nevertheless, we acknowledge that this research has two limitations. On the one hand, we had only studied the composition, diversity, and function of fungal communities in the wild and reintroduced plant of rhizospheres of *M. sinica*. On the other hand, this study had only explored the impacts of rhizosphere microorganisms on *M. sinica* plants. Therefore, we should fully consider the effects of plant compartments (root endosphere and phyllosphere) and non-rhizosphere soil on the wild and reintroduced *M. sinica* associated microbial communities in the future work. At the same time, in addition to the fungal community, we should also consider the composition, diversity, and function of other microbial communities, including prokaryotes (bacteria and archaea), even including protists and viruses. Furthermore, culture-dependent methods or a combination of multi-omics methods should also be considered for use in future research.

## 4. Materials and Methods

### 4.1. Collection of Samples

*M. sinica* is considered to be the most primitive of the Magnoliaceae and is an ideal landscaping and a high-quality timber species. Wild and reintroduced *M. sinica* samples were collected in Xichou County (23°37′03″–23°42′98″ N, 104°77’33″–104°82’72″ E), Wenshan Zhuang and Miao Autonomous Prefecture, Yunnan Province, with an average altitude from 1383 to 1605 m. The areas where the wild and reintroduced *M. sinica* were growing are approximately 5 km apart, with no connection between the two areas, and were in independent populations, with the same climatic conditions, including the same temperature, precipitation, and soil types. In July 2018, a survey of the diameter at breast height (DBH) of the plants was carried out in these areas, and three plants with the same DBH were randomly selected in each of the two areas, representing three replicates. The region (5 × 5 m) around each *M. sinica* individual was used as a plot; then, a soil knife was used to slowly dig out the fine roots and the basal parts of each individual. The rhizospheric soil of each individual was collected at five points along an “S” shape in each plot according to the shaking-off method [1]. The soil collected from the 5 points was packed into sterilized bags and thoroughly mixed as the rhizospheric soil from each plot. A total of six rhizospheric soil samples were collected, including three wild (WR1, WR2, and WR3, as three replicates of “WR”) and three reintroduced rhizospheric soil samples (RR1, RR2, and RR3, as three replicates of “RR”). All collected rhizospheric soil samples were divided into two parts, one of which was quickly returned to the laboratory (stored at a low temperature during transportation) and stored at −80 °C for fungal community diversity evaluation. The other part was ground and air-dried. These soil samples were passed through a 50-mesh sieve and stored in a self-sealing bag for the determination of soil physical and chemical properties.

### 4.2. Rhizospheric Soil Physicochemical Properties

The following physicochemical indexes were measured for the six soil samples in this study: soil pH (NY/T 1377-2007), OM content (NY/T 1121.6-2006), hydrolyzed nitrogen (HN), available potassium (AK, NY/T 889-2004), total nitrogen (TN, NY/T 53-1987), total phosphorus (TP, NY/T 88-1988), total potassium (TK, NY/T 87-1988), electrical conductivity (EC, HJ 802-2016), and total water-soluble salt (TWSS, NY/T1121.16-2006). The physicochemical properties mentioned above were measured by reference to the corresponding national standards [38]

### 4.3. Extraction of Genomic DNA from Rhizospheric Soil

The Power Soil Extraction Kit (Mo Bio Laboratories, United States) was used to extract the total DNA from each sample according to the kit’s instructions [24]. The integrity of the total DNA from each sample was observed using 2% agarose gel electrophoresis, and the concentration and purity of the DNA were determined with a NanoDrop 2000 spectrophotometer. The DNA concentration in all samples was higher than 30 ng/µL, and the values of OD_260_/OD_280_ were between 1.8 and 2.0. Therefore, the concentration and purity of all samples reached the ITS amplification requirements.

### 4.4. Amplification of the Internal Transcribed Spacer 2 (ITS2)-Region Sequences

The primers ITS3F (5′-GCATCGATGAAGAACGCAGC-3′) and ITS4R (5′-TCCTCCGCTTATTGATATGC-3′) were used for PCR amplification of the fungal ITS2 region from the wild and three reintroduced rhizosphere samples [39]. High-fidelity enzymes and specific primers were used in the PCR process in this study with Barcode. The following components were added to a sterile PCR tube: 5× FastPfu Buffer, 4 µL; 2.5 mM dNTPs, 2 µL; FastPfu Polymerase, 0.4 µL; BSA, 0.2 µL; soil sample DNA, 10 ng; the primer ITS3F (5 µM), 0.8 µL; the primer ITS4R (5 µM), 0.8 µL; sterile ddH_2_O up to 20 µL. The PCR amplification procedure was as follows: 3 min for denaturing at 95 °C, 37 cycles (30 s at 95 °C; 30 s for annealing at 53 °C; 45 s at 72 °C), 10 min for an extension at 72 °C, and storage at 10 °C for future study. Finally, amplicon sequencing was performed using the Illumina MiSeq PE 300 platform (Illumina, San Diego, CA, USA). The sequencing of all samples in this study was performed by the Shanghai Majorbio Bio-pharm Technology Co. Ltd. The raw sequences of six samples were deposited in the GenBank database (BioProject ID: PRJNA595467).

### 4.5. Data Analysis

FLASH (version 1.2.11) software was used to splice the reads from each sample, and high-quality clean tags were obtained [1]. The software QIIME (version 1.9.1) and Fastp (version 0.19.6) were used to filter the clean tags and obtain effective tags. Effective tags were then clustered into operational taxonomic units (OTUs) using a 97% similarity threshold, and the screening and classification of OTUs were completed with UPARSE (version 7.0.1090) software [25]. Species annotation was performed to compare the OTU sequences with those in the fungal ITS database in UNITE (version 8.0), and the relevant confidence threshold in the software was set to 0.7 [24]. The sequences from the six soil samples were subsampled based on the minimum number of sequences, and then the corresponding alpha and beta diversity values of the fungal communities were analyzed. The FUNGuild database was used to identify the ecological function categories of the rhizospheric fungi, OTUs with fewer than three sequences in the OTU list were eliminated, and the functional prediction and classification of the fungal communities were performed according to the FUNGuild database [23]. The data were processed with Excel 2013 and SPSS 20.0 software, and all statistical analyses in this study used a *t*-test to compare two groups of samples. The bioinformatics analysis was performed by using the free online Majorbio Cloud Platform (www.majorbio.com).

## 5. Conclusions

To the best of our knowledge, this study is the first to illustrate the composition, structure, diversity, and ecological functions of fungal communities in the rhizospheres of wild and reintroduced *M. sinica* as well as the correlations between soil physicochemical properties and these fungal communities. The compositions of the fungal communities in wild and reintroduced plant rhizospheres were very similar, while the relative abundances of fungal taxa differed significantly. The richness of the fungal communities in the reintroduced rhizospheric soil was higher than that in the wild plant rhizospheric soil, but the diversity of the fungal communities in the wild plant rhizospheric soil was higher than that in the reintroduced plant rhizospheric soil. Soil nutrients and litter significantly affected the diversity of the fungal communities in the wild and reintroduced plant rhizospheres. The physiological characteristics (DBH and plant height) of the *M. sinica* plants were also speculated to be important factors influencing the diversity of the fungal communities in the rhizospheric soil of wild and reintroduced *M. sinica*. There were a large number of antagonistic fungi in the wild plant rhizosphere, which represent an abundant microbial resource for biological control and the screening of antagonistic microorganisms.

## Figures and Tables

**Table 1:** Physical and chemical properties of the rhizospheric soil from wild and reintroduced *M. sinica.*

Sample ID	pH	Organic Matter (OM, g/kg)	Hydrolyzed Nitrogen (HN, mg/kg)	Available Potassium (AK, mg/kg)	Total Nitrogen (TN, g/kg)	Total Phosphorus (TP, g/kg)	Total Potassium (TK, g/kg)	Electrical Conductivity (EC, us/cm)	Total Water-Soluble Salt (TWSS, g/kg)
WR	4.55 ± 0.02A	145.24 ± 1.27B	545.58 ± 0.97A	268.72 ± 1.72B	7.45 ± 0.03A	0.89 ± 0A	23.24 ± 0.03A	157.3 ± 0.95B	1.25 ± 0.25A
RR	4.95 ± 0.01B	64.03 ± 1.6A	349.21 ± 0.97B	335.21 ± 2.89A	3.44 ± 0.04B	0.75 ± 0A	19.18 ± 0.07B	125.67 ± 0.65A	0.82 ± 0.26A

Different uppercase letters within the same column indicate extremely significant differences between wild and reintroduced *M. sinica* at *P* < 0.01. Mean ± SD (rhizospheric soil samples from wild plants (WR, *n* = 3); rhizospheric soil samples from reintroduced plants (RR, *n* = 3)).

**Table 2:** Alpha-diversity analysis of the fungal ITS2 region obtained from rhizospheric soil samples from wild and reintroduced *M. sinica*.

Sample ID	The Total Number of ITS Sequences	The Number of Valid Sequence of Each Sample	α-Diversity Index					
			Sobs	Shannon	Simpson	ACE	Chao	Coverage
RR1	70,870	67,730	488.00	3.30	0.14	648.85	635.00	0.996
RR2	35,598	34,720	328.00	1.99	0.27	553.41	522.24	0.996
RR3	73,514	69,900	590.00	3.38	0.10	731.44	721.68	0.995
RR: Mean ± SD	59,994 ± 21,168.88a	57,450 ± 19,714.64a	468.67 ± 132.07a	2.89 ± 0.78a	0.17 ± 0.09a	644.57 ± 89.09a	626.3 ± 100a	0.996 ± 0.001a
WR1	56,293	51,558	611.00	4.44	0.03	683.88	683.67	0.997
WR2	74,573	72,990	299.00	2.66	0.15	364.29	354.22	0.998
WR3	49,088	48,000	412.00	2.59	0.29	537.33	565.77	0.996
WR: Mean ± SD	59,984.67 ± 13,137.45a	57,516 ± 13,518.44a	440.67 ± 157.96a	3.23 ± 1.05a	0.15 ± 0.13a	528.5 ± 159.98a	534.55 ± 166.93a	0.997 ± 0.001a

Different uppercase letters within the same column indicate significant differences between wild and reintroduced *M. sinica* at *P* < 0.05. Mean ± SD (*n* = 3, WR1, WR2, and WR3 represent the three replicates of rhizospheric soil samples from wild plants (WR); RR1, RR2, and RR3 represent the three replicates of rhizospheric soil samples from reintroduced plants (RR)).
